# Laser Optical Feedback Turns 60

**DOI:** 10.3390/s23031176

**Published:** 2023-01-19

**Authors:** Maurizio Dabbicco, Lorenzo Luigi Columbo, Julien Perchoux

**Affiliations:** 1 Dipartimento Interateneo di Fisica “Michelangelo Merlin”, Università degli Studi di Bari “Aldo Moro”, via Amendola 173, IT-70126 Bari, Italy; 2 Dipartimento di Elettronica e Telecomunicazioni, Politecnico di Torino, corso Duca degli Abruzzi 24, IT-10129 Torino, Italy; 3 LAAS-CNRS, Université de Toulouse, CNRS, INP-ENSEEIHT, FR-31400 Toulouse, France

As soon as a laser is fired, some of the emitted light is scattered backward and coupled with the cavity modes, causing instability. However, already in March 1962, Kleinman and Kisliuk [1] suggested that controlled back reflection from an external mirror could help the stabilization of the fundamental cavity mode by suppressing the higher-order ones. Soon afterward, King and Steward [2] proposed the exploitation of optical feedback for metrology, and laser self-mixing (LSM) eventually became an established research topic. Sixty years and a few thousand publications later, this Special Issue celebrates some of the most recent achievements in optical feedback interferometry (OFI), as LSM is currently addressed.

The Special Issue includes four research articles, each covering one aspect of the multivariate system simply consisting of a laser and a scattering target. These papers relate to modeling new type of lasers, implementing commercial applications, and deepening our understanding of laser dynamics.

## Optical Feedback Hits Hard in THz QCL

In 1980, the seminal paper by Lang and Kobayashi [3] (which has received over three thousand citations) paved the way to understanding and modeling the rich dynamics of semiconductor lasers under optical feedback. In the following decades, much work has been conducted in refining the LK model and adapting it to special types of lasers, especially by the research groups of Petermann [4], Bosch [5], Brambilla [6] and Rakic [7]. The article by Qi et al. [8] included in the Special Issue solves the LK equations by relaxing the approximation, which is always made, of a single external cavity roundtrip. The authors study one class of semiconductor lasers currently at the forefront of laser research, the THz Quantum Cascade Laser (QCL), and ensure the occurrence of multiple reflections in the external cavity. In this strong feedback regime, THz QCLs exhibit self-pulsation which enables modulation-free (fixed bias) THz imaging.

## The Five-Feedback-Regimes Frame Gets a New Dimension

The very first experimental classification of the feedback-induced changes on laser emission was developed six years later the work of LK, by Tkach and Chraplyvy [9]. Their study is a milestone with almost one thousand citations and set the framework for later development, notably by Donati and Horng [10] and Jumpertz et al. [11]. The article published in this Special Issue by Bertling and co-workers [12] adds a new dimension to the diagram of the self-mixing regimes, typically framed by the target distance and the feedback strength axis. They studied the role of the laser bias current in setting the switching point between different operational regimes in two types of semiconductor diode lasers, which are mostly used in LSM applications: vertical cavity surface emitting lasers (VCSELs) and distributed feedback Bragg (DFB) lasers. The laser bias current is quite an important, as well as underestimated, parameter of the system, both because it is fully under the operator′s control and because it can be tuned to optimize the signal for a given feedback strength.

## Laser Self-Mixing Rangefinders Target Consumers with Sub-Millimeter Resolution

As soon as it was recognized that LSM in diode lasers can be used to measure arbitrary displacement without ambiguity [13], the method become a workhorse application of Optical Feedback Interferometry. The intrinsically better resolution achievable at a short wavelength by GaN blue lasers [14] has been improved upon by the extreme subwavelength resolution realized by the stability of THz QCLs [15]. Much more difficult for an interferometer is to measure the absolute distance, if not aided by an independent reference signal. The article published in the Special Issue by Cavedo et al. [16] opens a new perspective to cost effective LSM rangefinders, relying on multiple frequency modulation. Their system achieves 0.1 mm accuracy across the one-decade range from 0.2 to 2 m, making a significant step towards commercial application in the consumer market.

## Colored Optical Feedback Turns Down the Noise in Chaotic Lasers

Optical feedback always pushes the laser emission to change instantaneous values of frequency and power. A relatively high feedback power may even drive the laser into what is called a coherence collapse characterized by a ten- to hundred-fold increase in the linewidth and a complete loss of phase information. At first, it was an annoyance. However, in the 1990s, the proprieties of coherently generated chaotic light [17], different from those of thermally generated incoherent light, began to attract attention of researchers and the new field of random lasers [18] and chaos-based communication took off and it is still flying high [19]. The article by Rota-Rodrigo et al. [20] included in the Special Issue deals with a special random laser system, the Raman fiber laser, where the randomly distributed feedback along a few kilometers of optically pumped fiber determines the noise properties of the laser emission. They show how the Relative Intensity Noise (RIN) transfer from the pump to the random laser can effectively be controlled and reduced by spectrally selected optical feedback.

## References

[1] Kleinman D.A., Kisliuk P.P. (1962). Discrimination Against Unwanted Orders in the Fabry-Perot Resonator. Bell Syst. Tech. J..

[2] King P.G.R., Steward G.J. (1963). Metrology with an optical maser. New Sci..

[3] Lang R., Kobayashi K. (1980). External optical feedback effects on semiconductor injection laser properties. IEEE J. Quantum Electron..

[4] Schunk N., Petermann K. (1988). Numerical analysis of the feedback regimes for a single-mode semiconductor laser with external feedback. IEEE J. Quantum Electron..

[5] Plantier G., Bes C., Bosch T. (2005). Behavioral model of a self-mixing laser diode sensor. IEEE J. Quantum Electron..

[6] Columbo L.L., Brambilla M. (2014). Multimode regimes in quantum cascade lasers with optical feedback. Opt. Express.

[7] Kliese R., Taimre T., Bakar A.A.A., Lim Y.L., Bertling K., Nikolić M., Perchoux J., Bosch T., Rakić A.D. (2014). Solving self-mixing equations for arbitrary feedback levels: A concise algorithm. Appl. Opt..

[8] Qi X., Loh H.Y., Taimre T., Bertling K., Indjin D., Rakić A.D. (2022). Self-Pulsations in Terahertz Quantum Cascade Lasers under Strong Optical Feedback: The Effect of Multiple Reflections in the External Cavity. Sensors.

[9] Tkach R., Chraplyvy A. (1986). Regimes of feedback effects in 1.5-µm distributed feedback lasers. J. Light. Technol..

[10] Donati S., Horng R.H. (2012). The diagram of feedback regimes revisited. IEEE J. Sel. Top. Quantum Electron..

[11] Jumpertz L., Carras M., Schires K., Grillot F. (2014). Regimes of external optical feedback in 5.6 μ m distributed feedback mid-infrared quantum cascade lasers. Appl. Phys. Lett..

[12] Bertling K., Qi X., Taimre T., Lim Y.L., Rakić A.D. (2022). Feedback Regimes of LFI Sensors: Experimental Investigations. Sensors.

[13] Donati S., Giuliani G., Merlo S. (1995). Laser diode feedback interferometer for measurement of displacements without ambiguity. IEEE J. Quantum Electron..

[14] Hast J., Okkonen M., Heikkinen H., Krehut L., Myllylä R. (2006). Nanometer-scale displacement sensing using self-mixing interferometry with a correlation-based signal processing technique. Opto-Electron. Rev..

[15] Mezzapesa F.P., Columbo L.L., De Risi G., Brambilla M., Dabbicco M., Spagnolo V., Scamarcio G. (2015). Nanoscale Displacement Sensing Based on Nonlinear Frequency Mixing in Quantum Cascade Lasers. IEEE J. Sel. Top. Quantum Electron..

[16] Cavedo F., Esmaili P., Norgia M. (2022). Self-Mixing Laser Distance-Sensor Enhanced by Multiple Modulation Waveforms. Sensors.

[17] Mork J., Tromborg B., Mark J. (1992). Chaos in semiconductor lasers with optical feedback: Theory and experiment. IEEE J. Quantum Electron..

[18] Wiersma D.S., van Albada M.P., Lagendijk A. (1995). Random laser?. Nature.

[19] Spitz O., Herdt A., Wu J., Maisons G., Carras M., Wong C.W., Elsäßer W., Grillot F. (2021). Private communication with quantum cascade laser photonic chaos. Nat. Commun..

[20] Rota-Rodrigo S., Leandro D., Santarelli G., Lopez-Amo M., Ania-Castañón J.D. (2022). Effect of Linewidth on the Relative Intensity Noise in Random Distributed Feedback Raman Fiber Lasers. Sensors.

